# Frontal presentation of Alzheimer's disease: a series of patients with biological evidence by CSF biomarkers

**DOI:** 10.1590/S1980-57642013DN70100011

**Published:** 2013

**Authors:** Leonardo Cruz de Souza, Maxime Bertoux, Aurélie Funkiewiez, Dalila Samri, Carole Azuar, Marie-Odile Habert, Aurélie Kas, Foudil Lamari, Marie Sarazin, Bruno Dubois

**Affiliations:** 1 Université Pierre et Marie Curie Paris 6, Centre de Recherche de l'Institut du Cerveau et de la Moelle Epinière, UMR-S975, 47-83 bd de l'Hôpital, 75013 Paris, France. Inserm, U975, 47-83 bd de l'Hôpital, 75013 Paris, France. CNRS, UMR 7225, 47-83 bd de l'Hôpital, 75013 Paris, France 4 Institut du Cerveau et de la Moelle Epinière, ICM, 47-83 bd de l'Hôpital, 75013 Paris, France. Alzheimer Institute; Research and Resource Memory Centre; Centre de Référence des Démences Rares, Centre de Référence Maladie d'Alzheimer jeune, AP-HP, Pitié-Salpêtrière Hospital, 47-83 boulevard de l'Hôpital, 75013 Paris, France.; 2 Université Pierre et Marie Curie Paris 6, Centre de Recherche de l'Institut du Cerveau et de la Moelle Epinière, UMR-S975, 47-83 bd de l'Hôpital, 75013 Paris, France. Alzheimer Institute; Research and Resource Memory Centre; Centre de Référence des Démences Rares, Centre de Référence Maladie d'Alzheimer jeune, AP-HP, Pitié-Salpêtrière Hospital, 47-83 boulevard de l'Hôpital, 75013 Paris, France.; 3 Service de Médecine Nucléaire, AP-HP, Groupe hospitalier Pitié-Salpêtrière, F-75013, Paris, France.; 4 Department of Metabolic Biochemistry, Pitié-Salpêtrière Hospital, Paris, France.

**Keywords:** Alzheimer's disease, frontotemporal dementia, CSF biomarkers

## Abstract

Besides its typical amnesic presentation, focal atypical presentations of Alzheimer's disease (AD) have been described in neuropathological studies. These phenotypical variants of AD (so-called "atypical AD") do not follow the typical amnestic pattern and include non-amnestic focal cortical syndromes, such as posterior cortical atrophy and frontal variant AD. These variants exhibit characteristic histological lesions of Alzheimer pathology at *post-mortem* exam. By using physiopathological markers, such as cerebrospinal fluid markers, it is now possible to establish *in vivo* a biological diagnosis of AD in these focal cortical syndromes. We report a series of eight patients who were diagnosed with behavioural variant frontotemporal dementia based on their clinical, neuropsychological and neuroimaging findings, while CSF biomarkers showed an AD biological profile, thus supporting a diagnosis of frontal variant of AD.

## Abstract (pt)

Além da típica forma amnésica, apresentações focais atípicas da doença de Alzheimer (DA) foram descritas em estudos anatomopatológicos. Essas variantes fenotípicas da DA ("DA atípica") não seguem o padrão amnésico convencional e incluem síndromes corticais focais não amnésicas, tais como a atrofia cortical posterior e a variante frontal da DA. Essas variantes apresentam lesões histológicas características da DA ao exame patológico *post-mortem*. O uso de marcadores fisiopatológicos da DA, como os biomarcadores do líquido cefalorraquidiano, permite estabelecer *in vivo* um diagnóstico biológico de DA nessas síndromes corticais focais. Reportamos uma série de oito pacientes que foram clinicamente diagnosticados como portadores da variante comportamental da demência frontotemporal (de acordo com critérios clínicos, neuropsicológicos e de neuroimagem), mas nos quais a investigação dos biomarcadores do líquor mostrou um perfil biológico de DA, de modo que o diagnóstico da variante frontal de DA foi finalmente estabelecido.

## INTRODUCTION

Alzheimer's disease (AD) has been classically defined as a progressive amnestic neurodegenerative disorder with subsequent emergence of other cognitive and neuropsychiatric changes that impair activities of daily living.^1^ In typical presentations, patients with AD manifest early episodic memory deficit followed by various associations with executive, language and visuospatial deficits. The identification of this specific clinical and cognitive profile has been the core of the clinical diagnosis of AD, as established by the NINCDS-ADRDA criteria.^2^

In contrast to this typical amnestic profile, focal atypical presentations of AD have been described in neuropathological studies.^3-6^ These phenotypical variants of AD (so-called "atypical AD")^7^ do not follow the typical amnestic pattern and include non-amnestic focal cortical syndromes, such as posterior cortical atrophy and frontal variant AD. These variants exhibit characteristic histological lesions of Alzheimer pathology at *post-mortem* exam. Alzheimer pathology is indeed the most frequent pathological diagnosis associated with posterior cortical atrophy. By contrast, it is less frequently reported in patients presenting prominent behavioural deficits^3,5,8,9^ such as those observed in the behavioural variant frontotemporal dementia (bvFTD).

With the recent advances in physiopathological markers of AD, the underlying pathological process of AD may be identified *in vivo* in patients who present with an atypical clinical presentation. By using physiopathological markers, such as amyloid markers on neuroimaging and cerebrospinal fluid (CSF) markers, it is now possible to establish *in vivo* a biological diagnosis of AD in these focal cortical syndromes.^10-12^

Here we report a series of eight patients who were diagnosed with bvFTD based on their clinical, neuropsychological and neuroimaging findings, while CSF biomarkers showed an AD biological profile, thus supporting a diagnosis of frontal variant of AD.

## METHODS

We searched the database at the Memory and Alzheimer Institute of the Pitié-Salpêtrière Hospital for patients for whom a diagnosis of bvFTD had been established according to clinical criteria. From this series, we selected those patients with a "CSF AD biomarker profile". A "CSF AD biomarker profile" was defined as a P-Tau/Aβ_42_ ratio higher than 0.21, as this distinguishes AD from bvFTD with a high sensitivity (91.2%) and specificity (92.6%).^11^ All selected patients in this "frontal AD group" fulfilled the revised Lund-Manchester consensus criteria for bvFTD^13-15^ including: [1] a corroborated history of initial progressive decline in social interpersonal conduct and behavioral symptoms such as emotional blunting, apathy, reduced empathy, disinhibition, stereotypic behaviors, alterations in food preference and poor self-care; [2] the presence of dysexecutive difficulties at the neuropsychological exam; [3] anatomical magnetic resonance imaging (MRI) and/or single Photon Emission Computed Tomography (SPECT) disclosing frontal atrophy and/or blood hypoperfusion.

We did not include subjects who presented with the following: [1] clinical or neuroimaging evidence of focal lesions; [2] severe depression; [3] early impairment of praxis and spatial skills; [4] patients with language disorders characteristic either of progressive non-fluent aphasia or semantic aphasia; [5] severe cortical or subcortical vascular lesions, and [6] inflammatory, infectious or vascular diseases that could account for cognitive/behavioral impairment.

Clinical and neuropsychological data from patients with frontal AD were compared with three groups of subjects selected from the database of the Memory and Alzheimer Institute of the Pitié-Salpêtrière Hospital: [i] patients with typical amnestic AD (n=18), with CSF AD biological profile (P-Tau/Aβ_42_ ratio higher than 0.21); [ii] patients with bvFTD (n=18) that fulfilled the last revised diagnostic criteria for bvFTD^15^ and who had normal CSF biomarker profile (P-Tau/Aβ_42_ ratio lower than 0.21); and [iii] normal controls (n=18) selected according to the following criteria: Mini-Mental State Exam (MMSE) ≥27 and normal neuropsychological testing.^16^ Subjects from frontal AD, typical amnestic AD and bvFTD groups were matched for educational level and disease duration.

**Statistical behavioral analysis.** All statistical analyses were performed with the STATISTICA 5.5A software (© StatSoft, Tulsa, Oklahoma, USA). Descriptive statistics were used to characterize each group. The Mann-Whitney test was employed to compare differences in distributions between the "frontal AD" group and each of the other three groups (healthy controls, typical AD and bvFTD groups).

**Measurement of CSF biomarkers.** CSF samples were collected by lumbar puncture (LP) and analyzed for total Tau, Tau phosphorylated at threonine 181 (P-Tau) and Aβ_42_ with a double-sandwich enzyme-linked immunosorbent assay (ELISA) method (Innogenetics, Gent, Belgium) at the Metabolic Biochemistry Department of the Pitié-Salpêtrière Hospital, as previously described.^11^

For all patients, the biological and clinical data were generated during a routine clinical work-up and were retrospectively extracted for this study. According to French legislation, explicit informed consent was waived, as patients and their relatives had been informed that individual data might be used in retrospective clinical research studies.

## RESULTS

Eight patients (seven men, one woman) were selected according to the inclusion criteria for "frontal AD". Demographic, clinical and neuroimaging data for each patient are shown in Table 1, while neuropsychological data are presented in Table 2. The age of patients at the time of clinical evaluation varied between 49 and 76 years. The age at onset of symptoms varied between 48 and 73 years. No patient had familial antecedents of bvFTD, but there was a family history of AD for two patients (patients "MY" and "RD"). According to inclusion criteria, all frontal AD patients had abnormal P-Tau/Aβ_42_ ratio. Moreover, all frontal AD patients had reduced Aβ_42_ (<450 pg/mL) and high P-Tau (>60 pg/mL) levels.

**Table 1:** Demographic, clinical, neuroimaging and CSF data for each patient.

	AJP	CN	LD	MJJ	MY	PM	RD	TA
Sex	Male	Male	Male	Male	Male	Male	Male	Female
Age at evaluation (years)	68	68	56	72	76	62	57	49
Age at onset (years)	59	62	54	70	73	58	55	48
Family history of AD	No	No	No	No	Yes	No	Yes	No
Behavioral symptoms	Apathy, gluttony, collectionism, obsessions, neglect of personal self-care	Apathy, aggressive behavior, motor stereotypies, indifference, desinhibition	Motor stereotypies, aggressive behaviour, logopenia, anosognosia	Apathy, anosognosia, decline in social interpersonal conduct	Joviality, desinhibition, decline in social interpersonal conduct	Apathy, gluttony, aggressive behaviour, obsessions, neglect of personal self-care, logopenia	Joviality, desinhibition, decline in social interpersonal conduct, anosognosia	Affective indifference, apathy, social withdrawal
Brain MRI	Not performed (normal CT scan)	Global cortical atrophy	Frontal atrophy	Frontal atrophy	Global cortical atrophy	Global cortical atrophy	Global cortical atrophy	Frontal atrophy
SPECT	Marked frontal hypoperfusion	Marked frontal hypoperfusion	Marked frontal hypoperfusion	Marked frontal hypoperfusion	Marked frontal hypoperfusion	Marked frontal hypoperfusion	Marked frontal hypoperfusion	Marked frontal hypoperfusion
Aβ_42_	210	230	186	317	125	167	412	329
Tau	386	357	648	1064	504	1200	1200	1200
P-Tau	76	70	97	138	84.5	159	202	140
Tau/Aβ_42_	1.84	1.55	3.48	3.36	4.03	7.19	2.91	3.65
P-Tau/Aβ_42_	0.36	0.30	0.52	0.44	0.68	0.95	0.49	0.43

**Table 2:** Neuropsychological data for each patient.

	AJP	CN	LD	MJJ	MY	PM	RD	TA
MMSE ( /30)	16	22	10	19	26	10	17	21
Orientation time/space ( /10)	8	10	4	8	10	2	4	8
MATTIS ( /144)	121	126	108	77	NA	101	111	121
MATTIS - Attention ( /37)	35	36	33	29	NA	36	36	37
MATTIS - Initiation ( /37)	35	27	28	13	NA	17	23	28
MATTIS - Construction ( /6)	5	6	4	4	NA	5	5	6
MATTIS - Concepts ( /39)	30	36	26	12	NA	36	35	31
MATTIS - Memory ( /25)	16	21	39	19	NA	7	12	19
Memory: Encoding (FCSRT) ( /16)	10	10	3	NA	14	2	9	8
Total Free Recall (FCSRT) ) ( /48)	8	9	NC	NA	13	NC	0	8
Total (Free + Cued) Recall (FCSRT) ( /48)	27	18	NC	NA	41	NC	12	14
Verbal Span (Direct - Indirect)	5-3	5-4	4-2	4-2	5-4	4-3	5-4	6-4
Phonemic Fluency in 2 minutes	14	10	3	1	4	3	14	7
Category Fluency in 2 minutes	18	13	3	2	21	5	15	6
FAB ( /18)	13	14	5	2	13	7	8	13
Wisconsin ( /20)	3	6	NA	3	9	3	NA	9
Mini-SEA	11.7	21.7	NA	NA	18.8	13.5	NA	12.1
Gestural Apraxia	Absent	Absent	Absent	Absent	Absent	Absent	Absent	Absent

FAB: Frontal Assessment Battery; FCSRT: Free and Cued Selective Reminding Test; Mini-SEA: Mini version of the Social Cognition and Emotional Assessment; MMSE: Mini-Mental State Exam; NA: Data not available; NC: Not continued (the evaluation of episodic memory was not continued due to severe encoding deficits).

The most frequent behavioral signs among patients with frontal AD were apathy (5 out of 8 patients), obsessive stereotypies (4 out of 8), decline in social interpersonal conduct (3 out of 8), irritability/aggressive behavior (3 out of 8), binge eating (2 out of 8), and neglect of personal self-care (2 out of 8). At the onset of the disease, two patients had predominantly inert behavior presentation; three patients had a disinhibited profile, and three others had a mixed profile.

Scores on the MMSE differed significantly between frontal AD patients and healthy controls, with lower scores for frontal AD patients (Table 3). All scores on frontal tests were significantly lower in frontal AD patients as compared to healthy controls (Table 3). More specifically, four frontal AD patients had time-space disorientation at neuropsychological exam, while four had good orientation. All frontal AD patients had poor performance on working memory tests (verbal spans). Dysexecutive deficits were present in all patients. Five out of eight frontal AD patients were evaluated with the short version of the Social Cognition and Emotional Assessment (Mini-SEA);^17^ all these patients had severe deficits on theory of mind and facial emotion recognition tests. Five out of eight frontal AD patients had episodic memory impairment on the Free and Cued Selective Reminding Test (FCSRT),^18^ with the so-called "amnestic syndrome of the medial temporal type" (low free recall not normalized with cueing). Two frontal AD patients had severe encoding deficits that limited the evaluation of episodic memory by the FCSRT and one patient did not undergo episodic memory evaluation with the FCSRT. No frontal AD patients presented gestural apraxia.

**Table 3:** Clinical and neuropsychological data between groups.

	Frontal AD (n=8)	Controls (n=18)	AD (n=18)	bvFTD (n=18)
Age at evaluation (years)	63.5 (±8.9)	68.6 (±7)^NS^	64.8 (±9.6)^NS^	65.7 (±7.9)^NS^
Disease duration (years)	3.5 (±2.4)	NA	3.4 (±1.7)^NS^	3.2 (±1.6)^NS^
Sex (male/female)	7/1	10/8	9/9	9/9
Educational level (in years)	10.4 (±3.9)	12.2 (±2.3)^NS^	10.5 (±3.9)^NS^	9.8 (±4.3)^NS^
MMSE ( /30)	17.6 (±5.6)	29.6 (±0.6)*	22.2 (±2.9)**	23.3 (±3.6)**
Orientation time/space ( /10)	6.3 (±2.9)	9.4 (±2.3)*	8 (±1.9)^NS^	8 (±1.8)^NS^
MATTIS ( /144)	109.3 (±16.7)	143.3 (±1.5)*	NA	119.2 (±12.3)^NS^
MATTIS - Attention ( /37)	34.6 (±2.8)	37 (±0)**	NA	33.9 (±3.6)^NS^
MATTIS - Initiation ( /37)	24.4 (±7.4)	36.5 (±1.0)**	NA	27.4 (±5.6)^NS^
MATTIS - Construction ( /6)	5 (±0.8)	6 (±0)**	NA	5.6 (±0.7)^NS^
MATTIS - Concepts ( /39)	29.4 (±8.5)	39 (±0)*	NA	32 (±4.9)^NS^
MATTIS - Memory ( /25)	19 (±10)	24.8 (±0.5)^NS^	NA	19 (±3.7)^NS^
Memory: Encoding (FCSRT) ( /16)	8 (±4.2)	15.7 (±0.6)*	12.4 (±1.9)*	13.8 (±3)**
Total Free Recall (FCSRT) ( /48)	5.4 (±5.3)	33.8 (±7.9)*	12 (±5.3)**	16.9 (±5.2)*
Total (Free + Cued) Recall (FCSRT) ( /48)	16 (±14.6)	46 (±1.8)*	30.8 (±8.6)**	39.9 (±7.6)**
Verbal Span (Direct)	4.8 (±0.7)	5.5 (±0.7)**	4.8 (±1.3)^NS^	5.2 (±1.4)^NS^
Verbal Span (Indirect)	3.3 (±0.09)	4 (±0.6)^NS^	3.4 (±1.2)^NS^	3.1 (±0.9)^NS^
Phonemic Fluency in 2 minutes	7 (±5.1)	13.7 (±3.2)**	8.2 (±6.3)^NS^	6.6 (±3.8)^NS^
Category Fluency in 2 minutes	10.4 (±7.3)	19.1 (±4.5)**	13.8 (±4.8)^NS^	10.6 (±3.9)^NS^
FAB ( /18)	9.4 (±4.5)	16.9 (±0.9)*	13 (±2.3)**	11.6 (±3.3)^NS^
Wisconsin ( /20)	6 (±3)	19 (±1.1)*	NA	2.5 (±1.7)^NS^
Mini-SEA	24 (±16)	41.2 (±6.9)*	NA	26.2 (±3.4)**

FAB: Frontal Assessment Battery; FCSRT: Free and Cued Selective Reminding Test; Mini-SEA: Mini version of the Social Cognition and Emotional Assessment; MMSE: Mini-Mental State Exam; NA: Data not available. Comparison between frontal variant AD patients and other groups was performed using a non-parametric Mann-Whitney U-test with the following annotations:

NS Non significant vs frontal variant AD group.

* p<0.001.

** p<0.05

There was no significant difference between frontal AD and bvFTD groups for MATTIS scores, verbal spans, verbal fluencies (phonemic and category) and for Wisconsin score (Table 3).

As an illustrative example, we report the clinical vignette of one of the patients included in this study. Mrs. TA, a medical nurse aged 48 years, was referred to the Behavioral Unit of the Pitié-Salpêtrière Hospital in April 2010 for marked apathy, affective indifference and social withdrawal which had been evolving for approximately one year. The patient also had a history of reduced verbal output. Her husband did not report memory difficulties or spatial disorientation. The activities of daily living were globally preserved. A previous psychiatric referral led to a diagnosis of depression, and the patient was in use of antidepressants (venlafaxine). Her preceding medical history was unremarkable. There was no history of hallucinations, head trauma, neuroleptic medications, or alcohol/drug abuse. There was no family history of neurological diseases or dementia. The standard neurological examination was normal, without abnormalities of eye movement. She had no motor signs, no extrapyramidal syndrome, and no myoclonus.

Neuropsychological tests showed an impairment in global cognitive efficiency (MMSE 21/30 and MATTIS scale 121/144), without disorientation in time and space. The patient presented a severe dysexecutive syndrome, with attentional and working memory deficits. Mental flexibility and the abilities of conceptualizing and programming were severely impaired on the Trail Making Test (TMT),^19^ on the modified Wisconsin Card Sorting Test^20^ and on the copy of Rey complex figure.^21^ The patient had impaired performance on tests of theory of mind and of facial emotion recognition (mini-SEA). There was an episodic memory deficit characterized by a low free recall (free recall score=8/48) not normalized with cueing (total recall=14/48). The patient had no deficits on the execution of gestures from the limb apraxia battery.^22^ There were no signs of Bálint or Gertsmann syndromes.

Language assessment demonstrated that there was no reading or writing impairment, with preserved written language comprehension. Verbal fluencies were reduced, both in phonemic (only four "p" words in two minutes) and categorical modalities (only nine fruits in two minutes), with slight difficulty on the denomination task (74/80). There was no speech apraxia and no semantic deficit.

**Figure 1:**
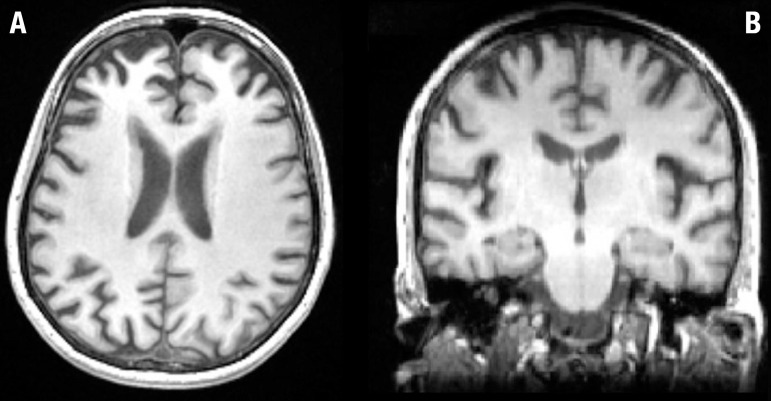
Brain MRI from patient TA, 48 year-old woman. [A] Axial slice: mild cortical frontal atrophy, predominant in medial regions. [B] Coronal slice: absence of hippocampal atrophy.

**Figure 2:**
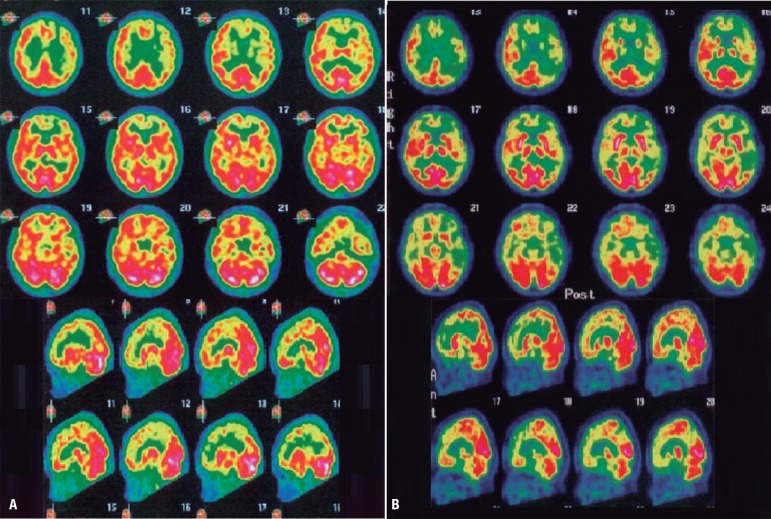
Brain scintigraphy (SPECT) from patient TA, 48 year-old woman. [A] Brain SPECT after approximately one year since symptoms onset (May/2010): moderate hypoperfusion in medial and dorsolateral prefrontal cortex, with left predominance; very mild hypoperfusion in the left parietal cortex; no hypoperfusion in medial temporal regions. [B] Brain SPECT approximately two years after symptoms onset (March/2011): severe hypoperfusion in prefrontal cortex (with left predominance); severe hypoperfusion in the left parieto-temporal cortex; mild hypoperfusion in the right parieto-temporal cortex; very mild hypoperfusion in the medial temporal regions.

Brain MRI (Figure 1) revealed mild cortical frontal atrophy, without medial temporal atrophy. Brain SPECT (Figure 2A) showed moderate hypoperfusion in medial and dorsolateral prefrontal cortex, with left predominance. There was very mild hypoperfusion in the left parietal cortex. Cerebral perfusion was preserved in medial temporal regions.

The patient underwent a complete blood and CSF exam in order exclude other causes of non-neurodegenerative dementia in young patients (autoimmune diseases, paraneoplastic pathology, CNS infection, metabolic diseases, and so on). These exams were all negative.

The diagnosis of bvFTD was initially established on a clinical basis by the neurologist (BD), as the clinical picture fulfilled the criteria for the disease: [1] a corroborated history of initial progressive decline in social interpersonal conduct, with apathy, affective indifference and loss of empathy; [2] the presence of severe difficulties in executive and social-emotional abilities; [3] atrophy of frontal lobes on brain MRI and marked hypoperfusion of frontal lobes on SPECT, with preservation of medial temporal and parietal regions. Taking into account these clinical data, the presence of significant amnesia was not considered incompatible with the diagnosis of bvFTD.

However, some weeks after hospitalization, data on CSF biomarkers were available and showed low Aβ_42_ (329 pg/mL), high Tau (1200 pg/mL) and high P-Tau (140 pg/mL), in favor of an AD diagnosis. All derived ratios (Tau/Aβ_42_ and P-Tau/Aβ_42_) were also in favor of AD. Considering these results, a diagnosis of frontal variant of AD was proposed.

On the clinical follow-up over 30 months, there was a marked deterioration in cognitive abilities and the patient manifested disorientation in time and space as well as limb apraxia and presented an aggravation of both amnesia and dysexecutive deficits. The patient clinically progressed to multi-domain cognitive impairment, with loss of autonomy, thus defining the dementia stage of the disease. The patient underwent another brain SPECT exam (Figure 2B) which showed severe hypoperfusion in prefrontal cortex (with left predominance), severe hypoperfusion in the left parieto-temporal cortex and mild hypoperfusion in the right parieto-temporal cortex. There was very mild hypoperfusion in the medial temporal regions. The patient has been treated with antidepressants and with an anticholinesterasic. She was also included in a clinical immunotherapy trial.

## DISCUSSION

We reported a series of eight patients which fulfilled clinical consensual criteria for bvFTD, but for whom a diagnosis of frontal variant AD was finally proposed on the basis of CSF biomarkers. Previous studies with either biological,^23^ genetic^24^ or pathological confirmation^3,5,8,25,26^ have also reported focal atypical presentations of AD mimicking bvFTD.

FTD is the second most frequent cause of degenerative dementia in patients below 65 years old^27^ and includes three clinical subtypes: the behavioral variant (bvFTD) and the language variants, progressive non-fluent aphasia and semantic aphasia.^27,28^

bvFTD is the most common presentation of FTD^29^ and is clinically characterized by an insidious and gradually progressive behavioral syndrome defined by a decline in social interpersonal conduct, impairment in regulation of personal conduct, emotional blunting and a loss of insight.^14^ bvFTD is typically associated with frontal and anterior temporal atrophy, in particular in the mesial and orbital prefrontal cortex, anterior insula and anterior cingulate cortices.^30^

From a pathological point of view, bvFTD belongs to the group of frontotemporal lobe degenerations (FTLD), which are characterized by a circumscribed atrophy of frontal and temporal cortex.^28^ FTLD have two major histopathological subtypes: FTLD with tau-positive inclusions (FTLD-tau), and FTLD with ubiquitin-positive and TAR DNA-binding protein (TDP) inclusions, but Tau-negative inclusions (FTLD-TDP).^28^ Alzheimer pathology is less frequently identified in patients clinically diagnosed as bvFTD.^3,5,8,9^ In agreement with these pathological data, it has been demonstrated that CSF biomarkers, which are considered surrogate markers of Alzheimer's pathophysiology, efficiently discriminate AD from bvFTD.^31^ In a previous study, we reported that only one out of 27 bvFTD patients presented a CSF AD biomarker profile.^11^

These pathological observations of AD presenting with symptoms that mimic bvFTD have led to the concept of "frontal AD".^8,25,26^ Besides this behavioral variant, AD may also present as other non-amnesic atypical focal variants, such as posterior cortical atrophy and logopenic aphasia. Taken together, these observations emphasize that not all patients with AD manifest a "typical" clinical pattern and that patients sharing a common pathology may be clinically heterogeneous.^6^

Conversely, pathologically different neurological disorders may share common symptomatology. This is the case for typical AD and bvFTD. For instance, apathy, a common feature of bvFTD, is also frequently observed in AD, even at initial stages of the disease.^32^ On the other hand, recent evidence has shown that marked amnesia, a hallmark of AD, is not uncommon in bvFTD patients. Episodic memory performance has been traditionally considered relatively preserved in bvFTD and amnesia was considered an exclusion criterion for the clinical diagnosis of bvFTD.^15^ However, it is increasingly recognized that bvFTD patients exhibit amnesia,^33-36^ even at early stages of the disease as up to 10% of pathologically-proven cases of bvFTD reported memory deficits.^37^ In a recent study, Hornberger, et al.^38^ analyzed the structural integrity of the memory circuit in AD and FTD *in vivo* and at *post-mortem*. Patients with FTD and AD patients did not differ on memory measures (visual recall with the Rey-Osterrieth Complex Figure Test, visual recognition with the Doors and People Test, and immediate recall from the Rey Auditory Verbal Learning Test). Moreover, they found that FTD and AD patients had similar degrees of hippocampal atrophy *in vivo*. More interestingly, they showed that FTD had more severe hippocampal atrophy at *post-mortem*. In line with these observations, neuroimaging studies have previously demonstrated that measures of hippocampal volumes do not accurately distinguish AD and bvFTD patients.^39-42^

Taken together, for diagnostic purposes, the reliance on exclusively phenotypical features assessed by "topographical markers"7, such as episodic memory deficits and hippocampal atrophy, may lead to misdiagnosis between AD and bvFTD. Until the development of biomarkers, the *in vivo* diagnosis of neurodegenerative dementias had been largely based on the identification of the presenting cognitive profile supported by neuroimaging. However, the diagnosis established according only to clinical "phenotypical criteria", without reference to an accurate biomarker, may lack confidence, as not all patients with dementia syndromes manifest a "typical" clinical pattern. Moreover, patients sharing a common pathology may be clinically heterogeneous and, conversely, pathologically different diseases may share common symptoms. The correspondence between clinical phenotype and underlying pathology is not always optimal.^5^ Accordingly, new proposals for diagnostic criteria of AD^7,43,44^ include "pathophysiological markers" such as CSF biomarkers for increased diagnostic efficiency.

The CSF is the optimal source of biological physiopathological markers, as it is in direct contact with the cerebral extracellular space.^45^ The neuropathological studies that analysed correlations of the levels of *in vivo* CSF biomarkers (total Tau [T-tau], phosphorylated Tau [P-Tau] and beta-amyloid peptide 1-42 [Aβ_42_]) with the intensity of the *post-mortem* cerebral lesions showed that CSF biomarkers predicted the presence of AD pathologic features with high accuracy.^46-50^ Considering these data, CSF biomarkers can be considered surrogate markers of AD-associated pathologic changes in the brain.^45,47-49^

The CSF levels of T-tau, P-Tau and Aβ_42_ or, even more specifically, the combination of low Aβ_42_ and high levels of T-tau and P-Tau, provide optimal sensitivity and specificity in the diagnosis of AD patients (even at MCI stage) against normal controls.^51,52^ The combined analysis of the CSF biomarkers, especially P-Tau/Aβ_42_ ratio, is also useful for the differential diagnosis between AD and frontotemporal lobar degeneration, regardless of its behavioural (bvFTD) or semantic presentation.^11,31^

CSF biomarkers or amyloid imaging may also identify patients with Alzheimer underlying pathology in atypical focal cortical presentations of AD, and thus may identify eligible patients for emerging anti-amyloid therapies.^10-12,23,53-57^ Including pathophysiological markers in the clinical investigation of patients with suspected progressive cognitive and behavioural disorders seems crucial given the prospect of disease-modifying drugs that can target the physiopathological process of neurodegenerative diseases.^58,59^

In this present series, all patients manifested typical bvFTD presentation and the diagnosis of atypical AD was possible only with CSF biomarker investigation. It is essential to use pathophysiological markers, especially in young subjects, in order to identify patients with atypical AD presentations and to propose a specific treatment for them.

While pathological data may be important for establishing diagnosis for patients, no autopsies were available in our cohort. It should be noted, however, that clinical diagnosis was established using accepted consensus criteria; all patients were extensively evaluated with clinical, biological, neuropsychological and neuroimaging exams at a center with expertise in the field of dementias. Furthermore, we selected patients with strict biological inclusion criteria based on CSF biomarker results, such as reduced Aβ_42_ level, high P-Tau and abnormal P-Tau/Aβ_42_ ratio, which have been demonstrated to be highly correlated with Alzheimer pathology at *post-mortem* exam.^47^

It would also be of value to compare the clinical and neuroimaging features across patients with frontal AD and bvFTD. Further studies with a greater number of patients are needed to investigate whether clinical, neuroimaging and neuropsychological parameters differ during disease progression of frontal AD and bvFTD.
